# Supplementary material for: A mixed-method analysis of provider adherence to integrated antenatal care guideline in BEmONC and Non BEmONC primary health center: An Indonesian case
Source: PLoS One. 2024 Aug 27;19(8):e0309454. doi: 10.1371/journal.pone.0309454 (PMC11349106; doi:10.1371/journal.pone.0309454)
Supplement: S1 Checklist — (DOCX) [file pone.0309454.s001.docx]

Table.... Mixed Method Reporting Checklist

| **No.** | **Mixed Method reporting checklist** | **Yes** | **N/E** | **NA** |  |
| --- | --- | --- | --- | --- | --- |
|  | **Assessment of the success of mixed methods studies in HSR** |  |  |  |  |
| 1 | Is the quantitative component feasible? | V |  |  | Quantitative Phase line 132-177 |
| 2 | Is the qualitative component feasible? | V |  |  | Qualitative phase line 179-210 |
| 3 | Is the mixed methods design feasible? | V |  |  | Study design line 110-119 |
| 4 | Have both qualitative and quantitative components been completed? | V |  |  | Result section consisting of qualitative and quantitative part. Line 218-356 |
| 5 | Were some quantitative methods planned but not executed? |  |  |  |  |
| 6 | Were some qualitative methods planned but not executed? |  |  |  |  |
| 7 | Did the mixed methods design work in practice? |  |  |  |  |
|  |  |  |  |  |  |
|  | **Assessment of the mixed methods design of studies in HSR** |  |  |  |  |
| 1 | Is the use of mixed methods research justified? | Yes |  |  | Abstract line 21-22  Method line 110 |
| 2 | Is the design for mixing methods described? |  |  |  |  |
|  | Priority | Yes |  |  | Line 11-114 |
|  | Purpose | Yes |  |  | Line 112-114 |
|  | Sequence | Yes |  |  | The quan precede qual (line110-111 |
|  | Stage of integration | Yes |  |  | Line 114-115 |
| 3 | Is the design clearly communicated? | Yes |  |  |  |
| 4 | Is the design appropriate for addressing the research questions? | Yes |  |  | To fully comprehend the implementation of integrated antenatal care, quantitative and qualitative data are both necessary. |
| 5 | Has rigour of the design been considered (proposal) or adhered to (report)? | Yes |  |  | We used Ochatain reporting checklist in preparation this paper. |
|  |  |  |  |  |  |
|  | **Assessment of the quantitative component of mixed methods studies in HSR** |  |  |  |  |
| 1 | Is the role of each method clear? | Yes |  |  | Quantitative Phase line 132-177  Qualitative phase line 179-210 |
| 2 | Is each method described in sufficient detail? | Yes |  |  | line 132-177 |
| 3 | Is each method appropriate for addressing the research question? | Yes |  |  | Line 88-90, and 112-114 |
| 4 | Is the approach to sampling and analysis appropriate for its purpose? | Yes |  |  | Line 145-148 |
| 5 | Is there expertise among applicants/authors? | Yes |  |  | Line 195-198 |
| 6 | Is there expertise on the team to undertake each method? | Yes |  |  | Line 195-198 |
| 7 | Have issues of validity been addressed for each method? |  | V |  |  |
| 8 | Has the rigour of any method been compromised? |  | V |  |  |
| 9 | Is each method sufficiently developed for its purpose? | Yes |  |  | line 132-177 |
| 10 | Is the (intended) analysis sufficiently sophisticated? | Yes |  |  | line 132-177 |
|  |  |  |  |  |  |
|  | **Assessment of the qualitative component of mixed methods studies in HSR** |  |  |  |  |
| 1 | Is the role of each method clear? | Yes |  |  | line 179-210 |
| 2 | Is each method described in sufficient detail? | Yes |  |  | line 179-210 |
| 3 | Is each method appropriate for addressing the research question? | Yes |  |  | Line 179-210 |
| 4 | Is the approach to sampling and analysis appropriate for its purpose? | Yes |  |  | Line 175-181 |
| 5 | Is there expertise among the applicants/authors? | Yes |  |  | Line 195-198, line 214-215 |
| 6 | Is there expertise on the team to undertake each method? | Yes |  |  | Line 195-198, line 214-215 |
| 7 | Have issues of validity been addressed for each method? | Yes |  |  | Line 194-197 |
| 8 | Has the rigour of any method been compromised? | Yes |  |  | Line 194-197 |
| 9 | Is each method sufficiently developed for its purpose? | Yes |  |  | Line 179-210 |
| 10 | Is the (intended) analysis sufficiently sophisticated? | Yes |  |  | Line 179-210 |
|  |  |  |  |  |  |
|  | **Assessment of integration in mixed methods studies in HSR** |  |  |  |  |
| 1 | Is the type of integration stated? | Yes |  |  | Line 114-115 |
| 2 | Is the type of integration appropriate to the design? | Yes |  |  | Line 114-116 |
| 3 | Has enough time been allocated for integration? | Yes |  |  | Line 23, Line 140-141 |
| 4 | Is the approach to integration detailed in terms of working together as a team? | Yes |  |  | Line 201-215 |
| 5 | Does the dissemination strategy detail how the mixed methods will be reported in final reports and peer-reviewed publications? |  |  | NA | This report is part of dissemination |
| 6 | Are the personnel who participate in the integration clearly identified? | Yes |  |  | Line 201-215 |
| 7 | Did appropriate members of the team participate in integration? | Yes |  |  | Line 201-215 |
| 8 | Is there evidence of communication within the team? | Yes |  |  | Line 201-215 |
| 9 | Has rigour been compromised by the process of integration? | Yes |  |  | Line 201-215 |
|  |  |  |  |  |  |
|  | **Assessment of the inferences made in completed reports of mixed methods studies in HSR** |  |  |  |  |
| 1 | Is there clarity about which results have emerged from which methods? | Yes |  |  | Result section consisting of quantitative part line 218-266 nd qualitative Line 267-357 |
| 2 | Are inferences appropriate? | Yes |  |  | Result section consisting of quantitative part line 218-266 nd qualitative Line 267-357 |
| 3 | Are the results of all the methods considered sufficiently in the interpretation? | Yes |  |  | Result section consisting of qualitative and quantitative part. Line 212-352 |
|  |  |  |  |  |  |
|  | NEI, not enough information; N/A, not applicable |  |  |  |  |
