# Supplementary material for: A mixed-method analysis of provider adherence to integrated antenatal care guideline in BEmONC and Non BEmONC primary health center: An Indonesian case
Source: PLoS One. 2024 Aug 27;19(8):e0309454. doi: 10.1371/journal.pone.0309454 (PMC11349106; doi:10.1371/journal.pone.0309454)
Supplement: Attachment — Submitted filename: respon to editor Plos one.docx [file pone.0309454.s004.docx]

PONE-D-23-12582
A mixed-method analysis of provider adherence to integrated antenatal care guideline in primary health centre: An Indonesian case

Dear Editor in chief,

Thank you for the review. We do apologize for being late in manuscript revision and fulfill the editorial requirement


1. Please complete all items on the Clinical Studies Checklist that are relevant for your submission, by following this link: <http://journals.plos.org/plosone/s/file?id=dc11/PLOSOne_Clinical_Studies_Checklist.docx> (

Thank you for your review, we have completed all clinical studies checklist as follow:

***PLOS ONE* Clinical Studies Checklist**

***PLOS ONE* manuscript number: _______________________**

| **Complete the following if your study involved human participants or human subjects’ data. These questions should be addressed for prospective and retrospective studies.** | | |
| --- | --- | --- |
| 1. | Did you obtain ethics approval for this study? - If yes, please upload (file type “Other”) the original approval document you received from your ethics committee. If the original document is in another language, please also provide an English translation. ___ Uploaded ___ N/A Our respons Yes, we obtained ethic approval for our study. And we have declared on line 177-179. We have added the explanation regarding ethical approval to clarify and addressed the plos one clinical study checklist. The sentences now amended: Ethics approval and consent to participate (line 174-187) This study was approved by the Medical/Health Research Bioethics Commission of the Universitas Islam Sultan Agung Islamic University Medical School (approval number 817/XII/2019/Komisi Bioetik). All methods were performed in accordance with the relevant guidelines and regulations under Bioethics commission of the Universitas Islam Sultan Agung research policy. A written informed consent was obtained from all participants on the four public health center in Semarang. Participants were informed of their right to withdraw from the study at any point and that the information obtained was confidential. For medical record review, patients’ names or hospital registration numbers were not used to ensure confidentiality, and access to participants’ information was given to researchers only. All recordings and transcripts were made anonymous before being discussed within the research group. Recorded in-depth interviews were then transcribed and coded by two coders (SY &HW). Thematic analysis was carried out systematically. Data from the in-depth interviews with the midwife coordinators were then triangulated with the document review, observation, and subsequent in-depth interviews with laboratory analysts and physicians were conducted. - If you did not obtain ethical approval, please explain why this was not required. |  |
| 2. | If your study involved human participants, please report in the Methods section when participants were recruited to the study.  ___ Completed ___ N/A  Our response  Yes, this study involve primary health care provider on the Qualitative phase, we have add the explanation in line 166-172 |  |
| 3. | If you are reporting a study of medical records or archived samples, please report in the Methods section the date range in which human subjects’ data/samples were collected and the date(s) when you conducted this study.  ___ Completed ___ N/A  Our response  Yes, this study reported medical record review regarding antenatal care procedure in primary health care care provider on the Qualitative phase (line 166-172). We have add the description in line 140-141 |  |
| 4. | Please specify in the Methods section whether authors had access to information that could identify individual participants during or after data collection.  ___ Completed ___ N/A  Our response  Thank you for your review. We have add the author access to participant individual information in line 184-188.. |  |
| 5. | If you are reporting an observational study – i.e. cohort, case-control, and cross-sectional studies – we recommend that the work is reported as per the requirements of the STROBE guidelines, and that you provide a completed STROBE checklist as a Supporting Information file with your submission.  The STROBE checklist was developed to improve the reporting of observational human subjects research, and is available here: <http://strobe-statement.org/fileadmin/Strobe/uploads/checklists/STROBE_checklist_v4_combined_PlosMedicine.docx>.  ___ Completed ___ N/A  Our response  Thank you for your careful review, this manuscript reporting a mixed method study, therefore we use Ochatain checklist as a guideline in reporting this study. |  |
| 6. | Please ensure that the author list and Corresponding Author entered in Editorial Manager match the author list and Corresponding Author in your manuscript file.  ___ Completed |  |

Our response

We have re-checked and ensured the list of authors and Corresponding Authors entered in Editorial Manager matches the list of authors and Corresponding Authors in your manuscript file.

There may be overlap between the checklist items and other queries listed below; please address any duplicated queries both in your response email and on the checklist itself. Upload the completed Clinical Studies Checklist as file type “Other” when you re-submit your manuscript. This document is for internal journal use only and will not be published if your article is accepted. The requested information will help us to assess whether your submission complies with PLOS ONE’s policies and adheres to applicable reporting standards. Note that your manuscript may be rejected if you provide incomplete or inadequate responses to the checklist questions and that changing the ‘Section/Category’ of your article does not affect this requirement.

2. Please provide additional details regarding participant consent. In the ethics statement in the Methods and online submission information, please ensure that you have specified (1) whether consent was informed and (2) what type you obtained (for instance, written or verbal, and if verbal, how it was documented and witnessed). If your study included minors, state whether you obtained consent from parents or guardians. If the need for consent was waived by the ethics committee, please include this information.

Our response:

Thank you for reminding me about this aspect. We have declared the informed consent for all participants in the Qualitative stage. And we did not take patient informed consent in the quantitative stage because we have only access to anonymous e-medical record recapitulation on demographic characterize and antenatal care procedure. Please see line 184-188.

If you are reporting a retrospective study of medical records or archived samples, please ensure that you have discussed whether all data were fully anonymized before you accessed them and/or whether the IRB or ethics committee waived the requirement for informed consent. If patients provided informed written consent to have data from their medical records used in research, please include this information

Our response:

Thank you for reminding me about this aspect. We have only access to anonymous e-medical record recapitulation on demographic characterize and antenatal care procedure. Please see line 184-188

Once you have amended this/these statement(s) in the Methods section of the manuscript, please add the same text to the “Ethics Statement” field of the submission form (via “Edit Submission”).

For additional information about PLOS ONE ethical requirements for human subjects research, please refer to <http://journals.plos.org/plosone/s/submission-guidelines#loc-human-subjects-research>.

Our response:

Thank you for reminding us about this. We have changed the ethical statement in the manuscript and made adjustments to the ethical statement in the submission form.

3. Please ensure that you include a title page within your main document. We do appreciate that you have a title page document uploaded as a separate file, however, as per our author guidelines (<http://journals.plos.org/plosone/s/submission-guidelines#loc-title-page>) we do require this to be part of the manuscript file itself and not uploaded separately.

Could you therefore please include the title page into the beginning of your manuscript file itself, listing all authors and affiliations.

Our response:

Thank you for your correction and I do apologize for my misunderstanding on separating file in title page. Now, we have compile the title page in the beginning of our text.
