# Supplementary material for: A mixed-method analysis of provider adherence to integrated antenatal care guideline in BEmONC and Non BEmONC primary health center: An Indonesian case
Source: PLoS One. 2024 Aug 27;19(8):e0309454. doi: 10.1371/journal.pone.0309454 (PMC11349106; doi:10.1371/journal.pone.0309454)
Supplement: Attachment — Submitted filename: Response to Reviewers.docx [file pone.0309454.s005.docx]

Thank you for submitting your manuscript to PLOS ONE. After careful consideration, we feel that it has merit but does not fully meet PLOS ONE’s publication criteria as it currently stands. Therefore, we invite you to submit a revised version of the manuscript that addresses the points raised during the review process.

Please submit your revised manuscript by Aug 10 2023 11:59PM. If you will need more time than this to complete your revisions, please reply to this message or contact the journal office at [plosone@plos.org](mailto:plosone@plos.org). When you're ready to submit your revision, log on to <https://www.editorialmanager.com/pone/> and select the 'Submissions Needing Revision' folder to locate your manuscript file.

Please include the following items when submitting your revised manuscript:

- A rebuttal letter that responds to each point raised by the academic editor and reviewer(s). You should upload this letter as a separate file labeled 'Response to Reviewers'.
- A marked-up copy of your manuscript that highlights changes made to the original version. You should upload this as a separate file labeled 'Revised Manuscript with Track Changes'.
- An unmarked version of your revised paper without tracked changes. You should upload this as a separate file labeled 'Manuscript'.

If you would like to make changes to your financial disclosure, please include your updated statement in your cover letter. Guidelines for resubmitting your figure files are available below the reviewer comments at the end of this letter.

If applicable, we recommend that you deposit your laboratory protocols in [protocols.io](http://protocols.io/) to enhance the reproducibility of your results. Protocols.io assigns your protocol its own identifier (DOI) so that it can be cited independently in the future. For instructions see: <https://journals.plos.org/plosone/s/submission-guidelines#loc-laboratory-protocols>. Additionally, PLOS ONE offers an option for publishing peer-reviewed Lab Protocol articles, which describe protocols hosted on [protocols.io](http://protocols.io/). Read more information on sharing protocols at <https://plos.org/protocols?utm_medium=editorial-email&utm_source=authorletters&utm_campaign=protocols>.

We look forward to receiving your revised manuscript.

Kind regards,

Phyllis Lau, PhD
Academic Editor
PLOS ONE

Journal requirements:

When submitting your revision, we need you to address these additional requirements.

1. Please ensure that your manuscript meets PLOS ONE's style requirements, including those for file naming. The PLOS ONE style templates can be found at

<https://journals.plos.org/plosone/s/file?id=wjVg/PLOSOne_formatting_sample_main_body.pdf> and

<https://journals.plos.org/plosone/s/file?id=ba62/PLOSOne_formatting_sample_title_authors_affiliations.pdf>

2. In your Data Availability statement, you have not specified where the minimal data set underlying the results described in your manuscript can be found. PLOS defines a study's minimal data set as the underlying data used to reach the conclusions drawn in the manuscript and any additional data required to replicate the reported study findings in their entirety. All PLOS journals require that the minimal data set be made fully available. For more information about our data policy, please see <http://journals.plos.org/plosone/s/data-availability>.

Upon re-submitting your revised manuscript, please upload your study’s minimal underlying data set as either Supporting Information files or to a stable, public repository and include the relevant URLs, DOIs, or accession numbers within your revised cover letter. For a list of acceptable repositories, please see <http://journals.plos.org/plosone/s/data-availability#loc-recommended-repositories>. Any potentially identifying patient information must be fully anonymized.

Important: If there are ethical or legal restrictions to sharing your data publicly, please explain these restrictions in detail. Please see our guidelines for more information on what we consider unacceptable restrictions to publicly sharing data: <http://journals.plos.org/plosone/s/data-availability#loc-unacceptable-data-access-restrictions>. Note that it is not acceptable for the authors to be the sole named individuals responsible for ensuring data access.

We will update your Data Availability statement to reflect the information you provide in your cover letter.

[Note: HTML markup is below. Please do not edit.]

Reviewers' comments:

Reviewer's Responses to Questions

**Comments to the Author**

1. Is the manuscript technically sound, and do the data support the conclusions?
The manuscript must describe a technically sound piece of scientific research with data that supports the conclusions. Experiments must have been conducted rigorously, with appropriate controls, replication, and sample sizes. The conclusions must be drawn appropriately based on the data presented.

Reviewer #1: Partly

Reviewer #2: Yes

2. Has the statistical analysis been performed appropriately and rigorously?

Reviewer #1: No

Reviewer #2: Yes

3. Have the authors made all data underlying the findings in their manuscript fully available?

The [PLOS Data policy](http://www.plosone.org/static/policies.action#sharing) requires authors to make all data underlying the findings described in their manuscript fully available without restriction, with rare exception (please refer to the Data Availability Statement in the manuscript PDF file). The data should be provided as part of the manuscript or its supporting information, or deposited to a public repository. For example, in addition to summary statistics, the data points behind means, medians and variance measures should be available. If there are restrictions on publicly sharing data—e.g. participant privacy or use of data from a third party—those must be specified.

Reviewer #1: No

Reviewer #2: Yes

4. Is the manuscript presented in an intelligible fashion and written in standard English?

PLOS ONE does not copyedit accepted manuscripts, so the language in submitted articles must be clear, correct, and unambiguous. Any typographical or grammatical errors should be corrected at revision, so please note any specific errors here.

Reviewer #1: No

Reviewer #2: Yes

5. Review Comments to the Author

Please use the space provided to explain your answers to the questions above. You may also include additional comments for the author, including concerns about dual publication, research ethics, or publication ethics. (Please upload your review as an attachment if it exceeds 20,000 characters)

Reviewer #1:

**Abstract.**

Please describe where and when the study was conducted. How do you assess the adherence?

Thank You for your careful review, for a better clarity, we have added the time, place and assessment checklist in the methods section of the abstract in the manuscript. (page 2 line 22-23)

**Introduction.**
Please provide the full name of CEmONC before put the acronym.

Thank You for your careful review, we have revised the introduction section, delete the sentence containing CEmNOC. We also revise the CEmNOC to CEmONC and move the sentence to the study setting and added the full name of the CEmONC as follow:

The government also requires every Comprehensive Emergency Obstetri and Newborn Care (CEmONC) hospital to regularly train their network of primary health care providers on maternal referral and emergency management based on the problem in referral cases. (page 4 line 98-99).

This sentence should be place in the methods: "Health providers in BEmNOC PHC administer parenteral drugs (i.e., antibiotics, uterotonic, oxytocin, and magnesium sulfate for preeclampsia and eclampsia). They also manually remove the placenta and retained products (e.g. manual vacuum extraction, dilation, and curettage), assisted vaginal delivery, and basic neonatal resuscitation."

Thank you for your review, the explanation of BEmNOC and non BEmNOC PHC role on providing health care service is important to explicitly show the difference in the role between the two PHC on antenatal care and delivery. Therefore this sentence should be mention on introduction rather than on method section. Hence, we have simplified and added the sentences better clarity:

The sentence previously was

Health providers in BEmNOC PHC administer parenteral drugs (i.e., antibiotics, uterotonic, oxytocin, and magnesium sulfate for preeclampsia and eclampsia). They also manually remove the placenta and retained products (e.g. manual vacuum extraction, dilation, and curettage), assisted vaginal delivery, and basic neonatal resuscitation.

The sentence now amended:

Basic Emergency Obstetric and Newborn Care (BEmONC) is an inpatient primary health care that includes 24 hours and seven days (24/7) of emergency maternal and newborn care ^3,16^. Recently, the World Health Organization (WHO) recommended achieving a minimum of eight ANC visits and the use of a core set of items of ANC services for safe motherhood^17^. Both types of PHC should provide a high-quality integrated ANC and adhere to the national minimum healthcare standards ^19^. A previous descriptive studies showed a difference in the achievement of minimum health care standards between the two types of PHC, with more women having antenatal care at least four (4^th^ ANC) visits in BEmONC than non-BEmONC PHC^20^. However, there was limited studies comparing the performance in delivering integrated ANC between BEmONC and non-BEmONC PHC. Thus, this study aims to describe the providers adherence to the Integrated ANC guidelines in BEmONC than non-BEmONC PHC and explore factors associated with the provider adherence. (Page 4 Line 81-90 )

Methods.
The 1st and 2nd paragraph of study setting should be part of the introduction as the rationale of the study.

Thank you for the review, I do agree with you that the two paragraphs should not be part of the method section. We have moved the two paragraphs from study setting to introduction section . (Page 3 Line 55-61 and line 73-80 )

Table 1 should be part of the result.

Thank you for the review, I do agree with you that the table 1 should not be part of the method section. We have deleted the table 1 from the method and moved it to the result section.

(Page 9 line 218-220)

There some redundancy of information between the study design section and the quantitative and qualitative section. Please select the information that you put there carefully, without repetition.

Thank You for a very careful review, we have identified the redundancy of the information between the two section and simplified them. We also added an explanation of the research data section written in this paper as follows:

This study employed an explanatory sequential mixed method design. The design was explanatory and sequential in that the quantitative phase preceded the qualitative phase. Both quantitative and qualitative data are presented equally to provide a comprehensive overview of the implementation of integrated antenatal care, as well as the various factors that influence it at both types of primary healthcare facilities. The integration of both quantitative and qualitative data was presented in the discussion section. This study was part of the participatory action research on the implementation of interprofessional collaboration in caring for pregnant women with heart diseases^29^ registered at the ISRCTN registry (<https://www.isrctn.com/ISRCTN82300061>)^30^. (page 5 line 110-118)

We also modified and added sentences on Quantitave and Qualitative section for better clarity.

Why do you choose medical records of ANC visits from November 2018 and August 2019 and not a 1-year period instead?

Thank you for your review we only included the medical record of ANC visit of the nine month due to technical matter. At the time of the study, there was migration from paper based to electronic medical record in the PHCs. We decided to include only the data from e-medical record to minimize the recall bias.

You stated that you select the PHC that collaborates with the hospital where the principal researcher works. Isn't that cause selection bias? How do you managed it?

Thank you for the review. I do agree with you in a sense that in the quantitative phase of our study this may cause selection bias, however it can be a strength for the qualitative part of our study. My position as a principal researcher working in the hospital collaborating with all PHC of the study setting can give a better credibility (one of method to achieve “trustwhortiness’. Although we did not use random sampling method we believe that the four selected PHC represent the characterize of the two different type of PHC (BEmONC and non BEmONC) and two different area (Suburban and urban)

You stated that "The provider adherence to integrated ANC guidelines was determined using the Indonesian Ministry of Health indicators enacted in 2012." However, in the result you also mentioned that you also analyzed whether it adhere to the local policy. Please also stated it in the methods.

Thank you for pointing this out, we have added the information about the use of the combination checklist (national and local integrated ANC guideline) in method as follow:

Providers adherence were evaluated using modified national integrated antenatal care guideline. The modified guideline was based on the national guideline with four additional items of Interdisciplinary examination and education. (Page 6 Line 150-152)

Please give more detail about the type of socio-demographic data collected.

Thank you for your careful review. We have added the socio-demographic data for a better clarity.

The sentence previous was:

The patient's socio-demographic information was descriptively analyzed

Now amended

The patient's socio-demographic information listed on medical record consisting of age, education level, obstetrical status, and the PHC status were descriptively analyzed (page 8 line 173-175)

In the results, besides Kruskal Wallis, you also use Chi-square and Fisher. Please stated it in the methods, and explain which data use which type of analysis. Also you must stated here that you analyzed the average for the continuous data, and conduct statistical analysis to identify the difference. Have you conducted normality test for numerical data?

Thank You for pointing this out, we do agree with your advise. We have added sentences to clarify the hypothesis test used. We also do apologize there is a typo on hypothesis testing used to analyze the differences of providers adherence to the integrated antenatal care guideline, written was the Kruskal-Wallis test, it should be Mann-Whitney test. We have added explanation of numerical data and we conducted Kolmogorov Smirnov normality test. We have mention all change in the revised sentence as follow:

Data were expressed as mean (standard deviation; SD), minimum-maximum, and number (n) and percentage (%) where appropriate. p<0.05 was considered statistically significant. Statistical analysis was made using IBM SPSS Statistics for Windows, Version 22.0 software (IBM Corp., Armonk, NY, USA). Fisher’s exact test (for 2×2 tables where some of the expected counts are less than 10), Likelihood Ratio (for bigger tables where 20% or more of the expected count is less than 5) and Pearson Chi-Square test were used to analyze the association of categorical variables, while the provider adherence score were analyzed using the non parametric Mann-Whitney U test (Kolmogorov-Smirnov test p=0,000). (Page 8 Line 176-183)

Please give more description regarding the SPSS, the company that produce it and the city where the company is located.

Thank you for your correction, we have change the sentence as requested.

Previous sentence was

Data analysis was carried out using IBM SPSS Statistics for Windows, Version 22.

The sentence now amended

Statistical analysis was made using IBM SPSS Statistics for Windows, Version 22.0 software (IBM Corp., Armonk, NY, USA). (Page 8 Line 178-179 )

Please also give more detail information regarding the "observation". Who conducted it? What is the instrument utilized? What is the qualification of the interviewer on the qualitative part? Since you stated that the PHCs were conveniently selected, how do you ensure that there is no interviewer bias?

Thank you for very detail review, we have added explanation regarding observation, qualification of the reviewer and trustwhortiness of the qualitative phase on the following paragraph:

Data from the in-depth interviews with the midwife coordinators were then triangulated with the document review (national and regional antenatal care policy), observation of the antenatal care facilities and three antenatal care consultations conveniently selected, and subsequent in-depth interviews with laboratory analysts and physicians. The interviews ranged between 30-40 minutes, and they were tape-recorded. Triangulation was carried out by SY assisted by research assistant (RA). She was involved in a 2015 qualitative study on interprofessional collaboration and has completed courses in qualitative research and good health research practice. RA is a fresh graduate medical doctor who helps the investigator technically with data collection. All documents, recordings, and transcripts were made anonymous before being discussed within the research group Colaizzi’s seven-step phenomenological approach was applied to the data analysis. The interviews were transcribed manually in Indonesia within 3x24 hours after the interviews by the research assistant. The two researchers cross-checked against the audio records. The entire data analysis process was conducted in Indonesian. The two researchers (SY, HW) applied the seven steps approach for the data analysis as follows: 1. to obtain a more accurate understanding of the descriptions, they read each transcript carefully, and combined with the field-notes taken from the interviews. 2. They extracted meaningful statements related to the participants’ perspective and the barriers of and promoting factors for provider’s adherence to the provider’s antenatal guideline. 3. They coded significant statements and labelled them with the participants’ keywords and phrases, and cross-checked and discussed all the codes. 4. They repeated and checked the first three steps, and each code was read, evaluated repeatedly, and deliberately before clustering them together into multidimensional categories. 5. They identified and described three sub-themes 6. They determined the final theme based on continuous discussion, comparison, reintegration, and inspection of the sub-themes. 7. They returned the transcript to participants to check their accuracy. HW is a master of public health who experiences as a facilitator of qualitative study courses. (page line 191-216)

**Results.**
The sentence, "These results showed that a compulsory education program and the minimum age of marriage law had been implemented well in this city22,33,34. It is belong to the discussion, not results.

Thank you for pointing this out. I have moved the sentence into the discussion section.

Table 1. The term "study participants" is not appropriate, as you only analyzed the medical records which is not requiring participation of the women.

Thank you for your correction, the term “study participant” was change to ‘pregnant women” to be more appropriate

Please also use complete full name for PHC and BEMNOC (not the acronym) and where is it located.

Thank you for your correction, we have add the full name and the location as requested

Although it is already narrated previously. A table should be self explained.

Thank you for pointing this out. We have added the explanation of acronym used in the table

The statement, "However, more pregnant women received an integrated ANC procedure in BeMNOC (average score of 42.26) than non-BEmNOC (average score 39.8)." This statement here reflect a proportion comparison, while you actually comparing the score, please rephrase.

Thank you for pointing this out. We have rephrased this sentence to be more appropriate as follows:

In Basic Emergency Obstetric and Newborn Care (BeMONC), a greater percentage of pregnant women (63.8%) received integrated ANC procedure in BeMONC PHC, compared to non-BeMONC (36.2%). There were no differences in provider adherence to the integrated ANC guideline between BEmONC and non-BEmONC PHC (p = 0.268). (Page 11-12 line 242-245)

The average score for integrated ANC procedure was higher (42.26) compared to non-BeMONC (39.8). (Page 14 line 266-267)

The statement, "The medical record review showed excellent documentation of interdisciplinary examination and education except for the first visits." This is actually an interpretation, which should be part of discussion. The result should only present fact.

Thank you for pointing this out. I have moved the sentence into the discussion section.

Table 3. You use "general state" while in the methods you use term "general condition". Please be consistent.

Thank you for your correction. We have selected and consistently use the term "the general physical examination" in this paper to describe the standard procedure for a physical examination.

Please use complete word for "and" not symbol "&". It appears in several parts of the manuscript.

Thank you for your correction, we've changed the & symbol to the word "and" as requested

For the p-value, please write "<0.001" for "0.00". The value is actually not zero, but the SPSS shortened it.

Thank you for your correction, we've changed the “0.00” to “<0.00 as requested (table 5 Page 14 Line 261)

The sentence, "Still, the interdisciplinary education was excellent in all PHC except for the dentist visit in the first visits." This is also discussion.

Thank you for pointing this out. I have moved the sentence into the discussion section.

You mentioned "reagent" several times. Please explain "reagent" for what.

Thank you for your correction. We have made an improvement on following sentences

Previous sentence:

Most participants mention the barrier of integrated ANC was limited time, too much patient and limited midwife and physician, and lack of laboratory reagent.

The sentence now amended

Several participants stated that the main obstacles to integrated ANC were limited time, a high number of patients, shortage of staff such as midwives and physicians, and insufficient diagnostic reagents, particularly for hepatitis B and HIV tests.

(Page 15 Line 279-281 )

We Also revised these sentence

Previous sentence

They mentioned that the possible reason for not adhering to the guideline was related to logistics, including lack of reagent and a manual record of reagent utilization leading to reagent stock out as follows:

The sentence now amended:

The laboratory analysts complained the problem with inventory of reagent especially on inventory control, they complaining of manual inventory of reagent leading to inaccuracy of the data of supplies affecting the availability of the stock to shortage of diagnostic reagent for antenatal care examination. Field observation showed that the laboratory care demand was 20 to 35 samples per day, run by one analyst with 7-8 working hours per day. (Page 15 line 295-299)

In the quote line 277, what is "general checking" refers to? Is it the same meaning with "general condition" or "general status"? Please be consistent in the terminology.

Thank you for your correction. We have selected and consistently use the term "the general physical examination" in this paper to describe the standard procedure for a physical examination.

You mentioned about policy document review. Please give more description in the methods.

Thank you for your correction, we have added the description of policy document review at method section (page 9 line 192)

Is BPJS-K the same with BPJS? Please be consistent.

Thank you for your correction. We have replace the “BPJS” to BPJS-K and move the explanation of BPJS in the first word.

Previous sentence was

In addition, it was partly due to the local government's lack of adherence to health service regionalization enacted by the BPJS. The local government policy stated that every citizen of Semarang could access health services throughout PHC in Semarang for free regardless of the regionalization rules of primary health facilities set by the BPJS.

Now amended

In addition, it was partly due to the local government's lack of adherence to health service regionalization enacted by the Health Social Security Administrative Bodies (Badan Penyelenggara Jaminan Sosial Kesehatan /BPJS-K). The local government policy stated that every citizen of Semarang could access health services throughout PHC in Semarang for free regardless of the regionalization rules of primary health facilities set by the BPJS-K. (Page 16-17 line 344-349)

For a better clarity we also add explanation of contact number indicators.

Previous sentence was:

There was a contradicting policy on regionalization of PHC among local government and the Health Social Security Administrative Bodies (Badan Penyelenggara Jaminan Sosial Kesehatan /BPJS-K). The local governance provides a free integrated ANC in all PHC for pregnant women with Semarang identity cards without regionalization. At the same time, BPJS-K limited the service performance assessment of PHC, especially the contact rate indicator, only counted on the patient who already registered as a member of BPJS in the PHC.

The sentences now amended:

There was a contradicting policy on regionalization of PHC among local government and the BPJS-K. The local governance provides a free integrated ANC in all PHC for pregnant women with Semarang identity cards without regionalization. At the same time, BPJS-K limited the service performance assessment of PHC, especially the contact rate indicator (the number of member of Indonesian Health Social Insurance visited to health facility or visited by the health provider), only counted on the patient who already registered as a member of BPJS in the PHC. (Page 16-17 line 350-357)

**Discussion.**
The statement (line 317), The different findings were related to the difference in assessment methods and content of the integrated ANC procedure. Explain more about the difference in the assessment methods.

Thank you for your careful review, we do agree to explain more the discrepancies in achievements were due to the differing assessment methods used. Our review of the instrument revealed that the ANC procedure content was evaluated differently. We have taken note of your feedback and added the explanation as follows:

Various findings were connected to the differences in assessment techniques including directly observing patients, reviewing medical records, and analyzing the content of integrated ANC procedures across different regions. (page 18 line 366-368)

Line 327: LMIC (Low Middle Income Country), should be the complete name first and the acronym in the bracket.

Thank you for your correction. We have rearrangged the term as requested (page19 line 390)

Line 333: Ministry of Health Regulation No. 97 of 2014, did you mean the Ministry of Health of Indonesia?

Yes, we mean in Indonesian. Thank you for your correction. We have change the sentence as follow:

Previous sentence

Ministry of Health Regulation No. 97 of 2014 emphasizes the provisions and authority of integrated ANC examination in both BEmONC and non-BEmONC PHC

Now amended

Regulation Of The Republic Of Indonesia Health Minister number 97 in 2014 emphasizes the provisions and authority of integrated ANC examination in both BEmONC and non-BEmONC PHC^21^ (page.19.line.395-397.)

Line 343: general perception assessment. what is "general perception assessment"? Please use same terminology through-out. Or provide explanation for each in the methods section.

Thank you for your correction. We have selected and consistently use the term "the general physical examination" to mention the the standard procedure for a physical examination and “general condition” to mention the result in this paper.

You should acknowledge the biases in your study methods as the limitations.

Thank you for your careful review we have add the limitation as follows:

In term of limitation, we included sample of four PHCs collaborates with the hospital where the principal researcher works, but it is however important to note that the selected PHC represent the characterize of the two different type of PHC (BEmONC and non BEmONC) and two different area (Suburban and urban). Do to the fact that we only evaluated the adherence based on the medical record there may be the problem in accuracy of the data for example the provider may not always record the procedur that they performed in medical record. Further study may needed to evaluate the adherence based on observation. (Page 22 line 352-358)

How is the generalizability of this study?

Thank you for pointing this out, I do agree to state the generalizability of this study, we have added the statement as follows:

Like most qualitative data, the findings are generalizable only to the primary health care of interest in the region of urban Indonesia. (Page 21 line 446-448)

**Conclusion.**
For the first statement please make clear that it is in Indonesia.

Thank you for your careful review, we have add the Indonesian to make clarity the conclusion as follows:

Previous sentence

The sentence now amended

Although the integrated ANC guideline was enacted by Indonesian ministry of health in 2014, few women in that four PHC In Semarang Indonesia (6%) received the full content of the integrated ANC.

The statement, "The average score of provider adherence to the integrated ANC guideline in non-BEmNOC PHC (66,66) was higher than BEmNOC (33,3). However, there was no statistical difference (p = 0.000)." It seems to not appear in the result previously. Conclusion should be based on the result. Where is the statement appear in the result? Please don't add new result in the discussion or conclusion.

Thank you for pointing this out. We do apologize for the typo in the average score in the conclusion section, the number has been revised accordingly. (page 21 line 463-464)

**Acknowledgement.**
Is The Indonesia Endowment Fund for Education funding the study? If so, please mention in the funding section.

The Indonesia Endowment Fund for Education funding did not funded this study but fund the doctoral program when this study was conducted, hence we delete that in acknowledgment. thank you for your correction. We have revised accordingly (page 22 line 472-474)

**General comment.**

The manuscript need to be proof-read by an English language editor.

Thank you for your advise, we went through the entire manuscript to eliminate grammatical mistakes and consulted it to the English language expert.

Also the author mentioned about reporting guidelines, but it is not attached.

Thank you for reminding me of this point. we have attached the reporting guideline for your reference.

Reviewer #2:

Dear Editor,

Thank you very much for the opportunity to review this manuscript, that the topic is close to my research interest and geographic expertise. The authors have provide quite comprehensive study about the adherence of antenatal care in Semarang Indonesia.

From me, there are couple of minor revisions that the authors need to address before the publication of this paper:

1. The author mentions BemNOC many times, which is I understand, However, I would suggest to use a more widely used Basic Emergency Obstetric and Newborn Care (BEmONC)-See UNFPA publication, to represent Puskesmas with PONED-an Indonesian term for it.

Thank you for your suggestion regarding the common acronym of BEmONC, we have changed the acronym as suggested.

2. Couple of abbreviations are not mentioned earlier but at the later part of the manuscript, for example LMIC-should be expand for the first time it is mentioned and BPJS-BPJSK.

Thank you for your correction. We have changed the word as requested

3. Details for data analysis, data collection, trustworthiness, demographic of qual participants for the qualitative interviews/FGs are missing.

Thank you for your careful review, we have added the data analysis on page 9 line 203-216

Data collection in.page 9 line 191-196

And demographic of qual participant as follows:

The demographic data of participant in in-depth interview of this study were :

| **Participant** | Participant 1 | Participant 2 | Participant 3 | Participant 4 | Participant 5 |
| --- | --- | --- | --- | --- | --- |
| **Age (year)** | 43 | 48 | 37 | 38 | 35 |
| **Working duration** | 22 | 24 | 15 | 17 | 15 |
| **Workplace** | PHC A | PHC B | PHC C | PHC D | PHD A |
| **Title** | midwife coordinator | midwife coordinator | midwife coordinator | midwife coordinator | Laboratory analyze |
| **Employment status** | permanent | permanent | permanent | permanent | permanent |

4. I would comment more also for the discussion and limitations, including implications for further research. This research would imply many, such as the need of training for non BEmONC, authority within primary care staff, and also perhaps further training for pregnancy complication screening-This was discussed superficially and authors discussed more on the numbers only.

Thank you for very careful review and also the suggestion sentences in the last part of discussion. We do agree to add more implication of our findings as follows:

The study revealed that healthcare providers in Semarang are not strictly adhering to the national integrated antenatal service guidelines. This is quite unexpected, especially since antenatal care coverage is as high as 98%. It is therefore crucial to reevaluate the criteria used to measure the success of ANC implementation, and not just focus on the quantity of care provided but also on its quality. These findings further support the research conducted in Tanzania, which revealed that the provision of ANC services was inconsistent and did not align with the recommended Focus Antenatal Care (FANC) guidelines^38^. (page 18-19, line 368-375)

We also added your suggestion in conclusion

This study recommends the need for regionalization of PHC work area, the need of training for non BEmONC including training of pregnancy complication screening, authority within primary care staff, and also intra- and inter-health centers collaborative care to provide the high quality of integrated antenatal care for every woman in Indonesia. (Page 22 line 466-470)

5. Implication for further research and limitation of the study, including limited number of women and health providers involved in the study have not been clearly acknowledged..
Thank you for pointing this out, I would improve the implication of the study and include your suggestion, in term of sample size, I agree with you that the we included limited number of participant, however the sample size has met the minimum number according to Hosmer-Lameshow formula.

While Semarang is one of the big cities in Indonesia that usually the availability at least one doctor in the daily PHC practice is usually warranted. It raised concerns on the quality of care rather than numbers.

Thank you for pointing this out, we have included your suggestion in the discussion section.

Thank you very much.

 [NOTE: If reviewer comments were submitted as an attachment file, they will be attached to this email and accessible via the submission site. Please log into your account, locate the manuscript record, and check for the action link "View Attachments". If this link does not appear, there are no attachment files.]

While revising your submission, please upload your figure files to the Preflight Analysis and Conversion Engine (PACE) digital diagnostic tool, <https://pacev2.apexcovantage.com/>. PACE helps ensure that figures meet PLOS requirements. To use PACE, you must first register as a user. Registration is free. Then, login and navigate to the UPLOAD tab, where you will find detailed instructions on how to use the tool. If you encounter any issues or have any questions when using PACE, please email PLOS at [figures@plos.org](mailto:figures@plos.org). Please note that Supporting Information files do not need this step.
