# Supplementary material for: A mixed-method analysis of provider adherence to integrated antenatal care guideline in BEmONC and Non BEmONC primary health center: An Indonesian case
Source: PLoS One. 2024 Aug 27;19(8):e0309454. doi: 10.1371/journal.pone.0309454 (PMC11349106; doi:10.1371/journal.pone.0309454)
Supplement: Attachment — Submitted filename: PONE.docx [file pone.0309454.s006.docx]

PONE-D-23-12582R1
A mixed-method analysis of provider adherence to integrated antenatal care guideline in primary health centre: An Indonesian case
PLOS ONE

Korvesi Vasiliki – peer review

The study reaches its aim to compare the provider’s adherence to the integrated antenatal care between two types of health care units. The structure is interesting but not innovative. Yet, maternal mortality reflects the quality of a health care system and therefore I have a positive opinion on this study.

Positive points:

Study design and methods are accurately described. The Statistical analysis used was adequate. Tables are clear and readable, table captions are complete and accurate.

Negative points:

The literature statistics should be in provided in table for easier studying.

I suggest a more detailed description of the local health care system. The structure of the health care system in developing countries and a brief description of the way it functions should be addressed.

In study design, lines 111-115 should be addressed in the results.

Language editing is recommended.

Confidential to the editors:

Due to lack of knowledge and experience working in a developing country health care system, I cannot fully understand the significance of this study. In my first opinion, I might have underestimated it. Yet after running the results, I reconsidered.
