# Supplementary material for: A mixed-method analysis of provider adherence to integrated antenatal care guideline in BEmONC and Non BEmONC primary health center: An Indonesian case
Source: PLoS One. 2024 Aug 27;19(8):e0309454. doi: 10.1371/journal.pone.0309454 (PMC11349106; doi:10.1371/journal.pone.0309454)
Supplement: Attachment — Submitted filename: PONE_ Response to Reviewers _28 Jan 2024.docx [file pone.0309454.s007.docx]

PONE-D-23-12582R1
A mixed-method analysis of provider adherence to integrated antenatal care guideline in primary health centre: An Indonesian case
PLOS ONE

Korvesi Vasiliki – peer review

The study reaches its aim to compare the provider’s adherence to the integrated antenatal care between two types of health care units. The structure is interesting but not innovative. Yet, maternal mortality reflects the quality of a health care system and therefore I have a positive opinion on this study.

Our response

Thank you for your careful review, to clarify the urgency of this article we have add the importance of this study on the background as follow.

Numerous studies have been conducted to identify the factors that contribute to maternal mortality rates, and one of these factors is the quality of antenatal care (ANC) provided. Although some studies have reported on the quality of ANC services, they have mainly focused on fulfilling the requirement of four visits during pregnancy. Only a few studies have evaluated adherence to national antenatal care guidelines. However, it is essential to note that the quality of ANC can be assessed by ensuring that all examination procedures are adequately fulfilled. (page 5 line 76-82)

We also add sentence to clarify the difference between BEmONC PHC and non-BEmONC as follow:

In Indonesia, there are two types of Primary Health Centers (Puskesmas): those with Basic Emergency Obstetric and Newborn Services (BEmONC) and those without. A BEmONC Primary Health Center provides additional inpatient and emergency services for mothers and newborns 24 hours a day, seven days a week6 (24/7) 3,37,38. Both types of PHC should provide a high-quality integrated ANC and adhere to the national minimum healthcare standards despite facility and staffing differences ^38,39^. (page 5 line 92-97)

Positive points:

Study design and methods are accurately described. The Statistical analysis used was adequate. Tables are clear and readable, table captions are complete and accurate.

Our response:

We greatly appreciate the feedback provided by our reviewers as it allowed us to significantly improve the quality of this writing. Their valuable input and directions helped us refine the content and make it more effective.

Negative points:

The literature statistics should be in provided in table for easier studying.

Our response:

Thank you for your kind review, We agree with your opinion, we have compiled several articles to strengthen the background and urgency of our research. As requested, we have presented the information in a table format for clarity. Page 3-4 Line 56-57

| Author, Source | setting and sample size | Study design | Summary of findings |
| --- | --- | --- | --- |
| Majrooh MA, Hasnain S, Akram J, Siddiqui A, Memon ZA.  Coverage and quality of antenatal care provided at primary health care facilities in the "Punjab" province of "Pakistan."  *PLoS One*. 2014;9(11). doi:10.1371/journal.pone.0113390 | 19 primary health care facilities of public sector (seventeen Basic Health Units and two Rural Health Centers were randomly selected from each district | Mixed Method  Quantitative and Qualitative methods | The overall enrollment for antenatal checkup was 55.9% and drop out was 32.9% in subsequent visits. The quality of services regarding assessment, treatment and counseling was extremely poor. The reasons for low coverage and quality were the distant location of facilities, deficiency of facility resources, indifferent attitude and non-availability of the staff. Moreover, lack of client awareness about importance of antenatal care and self-empowerment for decision making to seek care were also responsible for low coverage. |
| Seyoum T, Alemayehu M, Christensson K, Lindgren H. Client Factors Affect Provider Adherence to Guidelines during First Antenatal Care in Public Health Facilities, Ethiopia: A Multi-Center Cross-Sectional Study.  *Ethiop J Health Sci*. 2020;30(6):903-912. doi:10.4314/ejhs.v30i6.8 | Public Health Facilities, Ethiopia  834 study participants  **Providers’ adherence**: Providers’ adherence  was defined as providers’ “conformity to,  fulfilling standard ANC guidelines as per the  national protocols | Cross- sectional study | The proportion of clients who received the complete provider’s adherence to the first antenatal care guideline was 32.25% (95% CI: 29.1-35.5). The mean adherence score was 16.78%. Women who had prior history of pregnancy and/or birth related  complications (AOR = 1.58; 95%CI: 1.04-2.04) and late antenatal care booking at gestational week 16 or greater (AOR =  1.45; 95%CI: 1.03-2.03) were significantly associated with clients receiving complete providers’ adherence to the first antenatal guideline |
| Benova L, Tunçalp Ö, Moran AC, Campbell OMR.  Not just a number: Examining coverage and content of antenatal care in low-income and middle-income countries.  *BMJ Glob Health*. 2018;3(2):1-11. doi:10.1136/bmjgh-2018-000779 | 10 LMICs  analyse ANC related to women’s most recent live birth up to 3 years preceding the survey | Descriptive Demographic and Health Survey | In all 10 countries, the majority of women in need of ANC reported 1+ ANC visits and over two-fifths reported 4+ visits. Receipt of the six routine components varied widely; blood pressure measurement was the most commonly reported component, and urine test and information on complications the least. Among the subset of women starting ANC in the first trimester and receiving 4+ visits, the percentage receiving all six routinely measured ANC components was low, ranging from 10% (Jordan) to around 50% in Nigeria, Nepal, Colombia and Haiti.  even among women with patterns of care that complied with global recommendations, the content of care was poor |
| Mansur AMSA, Rezaul KM, Mahmudul HM, S C.  Quality of antenatal care in primary health care centers of bangladesh.  *J Family Reprod Health*. 2014;8(4):175-181. | purposively selected three upazilas among the clients receiving antenatal care (ANC).  Data were collected with questionnaire cum checklist in the context of two aspects of quality issues, namely assessment of physical arrangements for ANC (input) and services rendered by the providers (process). | descriptive cross sectional | Out of 528 multigravid respondents 360 (68.2%) took ANC in their previous pregnancy whereas 168 (31.8%) did not take ANC Pregnancy outcome was found to be associated with receiving ANC (χ2=73.599; p=0.000). Respondents receiving ANC had more good pregnancy outcome. The mean waiting time for receiving ANC was 0.77±.49 hours. Out of the 13 centers, only 3 (23.1%) have sufficient instruments to render ANC services. Findings showed that where the modes of ANC service delivery in the ANC centers are fairly satisfactory. Though some of the points of standard operation procedures (SOPs) on ANC are not covered by some ANC centers, those were not considered necessary. But, regarding the physical facilities available for rendering ANC services, it is seen that facilities are not quite satisfactory. Number of doctors and nurses are not very satisfactory. One of the centers under this study has no doctor, where ANC services are given by nurses.  most of the services and advice were received by more than 60% of the respondents. |

I suggest a more detailed description of the local healthcare system. The structure of the health care system in developing countries and a brief description of the way it functions should be addressed.

Our response:

Thank you for your thorough review. We have taken note of the inadequate explanation provided earlier regarding the health service system for antenatal care. To address this, we have updated the study setting section on page 7, lines 127-147, with a more comprehensive and detailed explanation.

The explanation is:

The maternal mortality rate in Indonesia has consistently shown a decreasing trend since 1991, dropping from 390 to 228 in 2011. Despite a brief increase to 359 in 2015, it went down again to reach a low of 189 in 2020^42^. In Semarang City, the maternal mortality rate has been on a continuous decline, with a decrease from 128 in 2015 to 71.35 in 2020, successfully achieving the national target. However, there was a concerning spike in 2021, with a rise to 95.30, but the number of maternal deaths decreased significantly to 67.25 in 2022^41,43^. Although cases of maternal mortality have decreased in Semarang City, this issue still requires special attention since the city of Semarang is the capital of Central Java Province, which sets an example for the surrounding areas and the entire province. To reduce maternal mortality, the Semarang City Government is implementing one program called SAN PIISAN (SAyaNgi and damPingi Ibu & Anak SemarANg City / caring and assisting mothers and Children in Semarang City), which is a comprehensive health program that assists individuals during the first 1000 days of life, starting from teenagers, prospective brides, pregnant women, giving birth, post-partum, infants, and toddlers under three months of age. The service-oriented paradigm prioritizes services based on needs and involves various stakeholders through #movingtogether. Additionally, the SAN PIISAN Innovation Program helps identify causes of maternal and infant mortality, such as pregnant women who are too young (<20 years), too old (>35 years), have more than four children, have had less than four previous pregnancies, and three delays in referrals. This innovation has been implemented since 2017 and continues to this day with the goal of reducing maternal and infant mortality rates.

In study design, lines 111-115 should be addressed in the results.

Our response

Thank you for bringing this issue to our attention. We have made the necessary changes to the results section of our study as per your request. We have included an explanation of the study design and presented an equal balance of both quantitative and qualitative data to provide a comprehensive overview of the implementation of integrated antenatal care. We have also discussed the various factors that have influenced the implementation of this care in both types of primary healthcare facilities. The changes primarily involve the integration of both qualitative and quantitative data in the results section. Page 13-22 line 278 -428

Language editing is recommended.

Our response

Thank you for your valuable feedback. We have taken immediate action and consulted with an English editor provided by our institution to make the necessary revisions to the manuscript.

Confidential to the editors:

Due to lack of knowledge and experience working in a developing country health care system, I cannot fully understand the significance of this study. In my first opinion, I might have underestimated it. Yet after running the results, I reconsidered.

Our response

We greatly appreciate the opportunity to receive constructive feedback on our writing through this review process. With the invaluable guidance of the reviewers, we are confident that our articles will be significantly improved and deemed worthy of publication. Thank you for the chance to enhance our skills and knowledge in this field.

Thank you for submitting your manuscript to PLOS ONE. After careful consideration, we feel that it has merit but does not fully meet PLOS ONE’s publication criteria as it currently stands. Therefore, we invite you to submit a revised version of the manuscript that addresses the points raised during the review process.

Our response

We appreciate your review of our article and reminder to adhere to author guidelines. We have made required revisions, and this manuscript will hopefully be suitable for publication.

==============================
**ACADEMIC EDITOR:**

- If the aim of the study is to compare BeMONC and non-BeMONC centers, please indicate so in the title

Our response

We appreciate your recommendation to add BEmONC in the manuscript title to make it more informative. We fully agree with your suggestion, and change the title as follow:

Previous Title

A mixed-method analysis of provider adherence to integrated antenatal care guideline in primary health centre: An Indonesian case

Now amended

A mixed-method analysis of provider adherence to integrated antenatal care guideline in BEmONC and Non BEmONC primary health center: An Indonesian case

- The authors mentioned that this is a larger study with PHCs caring for women with heart diseases. Does this mean this study includes only women with heart diseases? Please elaborate

Our response

Thank You for your thorough review, yes, this research is a part of a larger study on interprofessional collaboration in pregnant women with heart defects. However, the data being reported is from preliminary research aimed at gaining an understanding of the antenatal care service system at the Community Health Center as a whole, rather than just focusing on pregnant women with heart defects. To collect this data, we applied for a separate ethical approval, which was different from the ethical approval obtained for the larger study.

ethical permission of the larger study entitle “study of participatory action research: integrated care pathway for pregnant women with heart disease in Indonesia”

The Ethical approval for this study have declared on page 9 Line 178-181,

The protocol of this study was approved by the Medical/Health Research Bioethics Commission of the Universitas Islam Sultan Agung (approval number 817/XII/2019/Komisi Bioetik). All methods followed the relevant guidelines and regulations under the Bioethics Commission of the Universitas Islam Sultan Agung research policy.

**- "**Adherence to guidelines was assessed based on the fulfillment of items in the modified national integrated ANC guideline checklist" --> What is the specific criteria? Does the failure of not checking one item is already considered non-adherence?

Our response

Thank you for reminding me of this important information. As per the National Modified Integrated Antenatal Care guidelines, it is necessary to complete all procedures without any omissions. Failure to carry out even a single procedure would result in non-compliance with the modified national integrated ANC guidelines. Furthermore, in our study, we analyzed the attainment of integrated ANC compliance scores in two types of PHCs: BEmONC and non-BEmONC. We presumed that BEmONC would have higher compliance scores than non-BEmONC. We have addressed the issue in question in our statement on page 10, lines 206-217.

- What is the estimated sample size for the qualitative phase? Why were only five women included?

Thank you for reviewing our explanation. We chose the coordinator midwife as they have a better understanding of the implementation of integrated antenatal care. This is because they are usually chosen for training, so it is assumed that the coordinator midwife at the primary health center can provide a more comprehensive answer regarding health provider compliance with the modified antenatal care national guideline.

- What is the MMR in Semarang over time compared to the national level?

Our response

Thank you for reminding me about the presentation on maternal mortality rates in Indonesia. We agreed to compare the MMR data in the city of Semarang, which is now included on page 7, lines 127-133.

The maternal mortality rate in Indonesia has been decreasing since 1991, going down from 390 to 228 in 2011. Although it increased to 359 in 2015, it decreased again to reach its lowest figure of 189 in 202042. Similarly, in Semarang City, the maternal mortality rate has been consistently decreasing from 128 in 2015 to 71.35 in 2020, successfully achieving the national target. However, there was an alarming spike in 2021, with the rate increasing to 95.30, but fortunately, the number of maternal deaths significantly decreased to 67.25 in 202241,43.

- In Line 464, the conclusion mentions no statistical difference despite the p value being less than 0.000 (which cannot be true anyway). Please explain

Our response

We appreciate you pointing out the typo and we have taken immediate action to correct it. Thank you for bringing this to our attention.

Previous sentence

However, there was no statistical difference (p = <0.000). Page 22 line 463-464

Now amended

However, there was no statistical difference (p =0.268). Page 26 Line 539-540

- In Line 242, the authors mentioned there were no differences, when it is more accurate to state that there is no statistically significant differences, as there are actually differences in the percentages.

Our response

Thanks for sharing this information. We use two methods to analyze the situation. The first method is to assess compliance by checking if all the modified National Guidelines for Integrated Antenatal Care have been followed without skipping any procedure. The second method is to calculate and compare the compliance scores of two health centers - BEmONC and non-BEmONC.

The two analyses had different results. For the first method, both types of health centers were equally non-compliant, so there was no significant difference (P=0.268). However, the second method, which analyzed compliance with the integrated ANC guideline, showed significant differences between the two types of health centers (p=0.000).

To clarify this issue, we have revised and added the sentence as follow:

Previous sentence

There were no differences in provider adherence to the integrated ANC guideline between BEmONC and non-BEmONC PHC (p = 0.268). –Page 11 line 242

Now amended.

The study also found no significant difference in healthcare providers' adherence to modified integrated ANC between two types of PHCs (BEmONC and non-BEmONC; p=0.268). Both types of community PHCs failed to perform all integrated ANC procedures for all pregnant women visiting ANC. However, upon quantitative analysis, there was a difference in healthcare providers' adherence scores (p=0.000) to the modified integrated ANC guidelines between BEmONC and non-BEmONC PHCs (Table 6). In Basic Emergency Obstetric and Newborn Care (BEmONC) greater percentage of pregnant women (63.8%) received integrated ANC procedures in BEmONC PHC compared to non-BEmONC (36.2%) (Table 5 and Table 6).

- I suggest the authors to use a proofreading service as there are careless typos all over the manuscript. The most egregious one is in line 123 with "agshsus", which I could not make neither heads nor tails what that mean.

Our response

Thanks to your diligent review, we have successfully eliminated all typos in this manuscript and have implemented proofreading services as a standard practice at our institution.

Previous sentence :

Data agshsusThe protocol of this study was approved by the Medical/Health Research Bioethics Commission of the Universitas Islam Sultan Agung (approval number 817/XII/2019/Komisi Bioetik).

Now amended:

The protocol of this study was approved by the Medical/Health Research Bioethics Commission of the Universitas Islam Sultan Agung (approval number 817/XII/2019/Komisi Bioetik). (page Line 178-180)

A rebuttal letter that responds to each point raised by the academic editor and reviewer(s). You should upload this letter as a separate file labeled 'Response to Reviewers'.

- A marked-up copy of your manuscript that highlights changes made to the original version. You should upload this as a separate file labeled 'Revised Manuscript with Track Changes'.
- An unmarked version of your revised paper without tracked changes. You should upload this as a separate file labeled 'Manuscript'.

[Note: HTML markup is below. Please do not edit.]

Reviewers' comments:

Reviewer's Responses to Questions

**Comments to the Author**

1. If the authors have adequately addressed your comments raised in a previous round of review and you feel that this manuscript is now acceptable for publication, you may indicate that here to bypass the “Comments to the Author” section, enter your conflict-of-interest statement in the “Confidential to Editor” section, and submit your "Accept" recommendation.

Reviewer #2: (No Response)

Reviewer #3: All comments have been addressed

2. Is the manuscript technically sound, and do the data support the conclusions?

The manuscript must describe a technically sound piece of scientific research with data that supports the conclusions. Experiments must have been conducted rigorously, with appropriate controls, replication, and sample sizes. The conclusions must be drawn appropriately based on the data presented.

Reviewer #2: Yes

Reviewer #3: Yes

3. Has the statistical analysis been performed appropriately and rigorously?

Reviewer #2: Yes

Reviewer #3: Yes

4. Have the authors made all data underlying the findings in their manuscript fully available?

The [PLOS Data policy](http://www.plosone.org/static/policies.action#sharing) requires authors to make all data underlying the findings described in their manuscript fully available without restriction, with rare exception (please refer to the Data Availability Statement in the manuscript PDF file). The data should be provided as part of the manuscript or its supporting information, or deposited to a public repository. For example, in addition to summary statistics, the data points behind means, medians and variance measures should be available. If there are restrictions on publicly sharing data—e.g. participant privacy or use of data from a third party—those must be specified.

Reviewer #2: Yes

Reviewer #3: Yes

5. Is the manuscript presented in an intelligible fashion and written in standard English?

PLOS ONE does not copyedit accepted manuscripts, so the language in submitted articles must be clear, correct, and unambiguous. Any typographical or grammatical errors should be corrected at revision, so please note any specific errors here.

Reviewer #2: Yes

Reviewer #3: No

6. Review Comments to the Author

Please use the space provided to explain your answers to the questions above. You may also include additional comments for the author, including concerns about dual publication, research ethics, or publication ethics. (Please upload your review as an attachment if it exceeds 20,000 characters)

Reviewer #2: Unfortunately, it is still unclear to me in this version, as only one highlight in the manuscript so I don't really know which one has been edited. Could the author provide the tracked changes all of the edits? or highlight it?

At the response to the reviewers also do not specifically answering the reviewers comment.

Our response

Thank you for your very thorough review. Actually, we have response all of the comment in the previous round. Unfortunately, we provide the traked changes on the other file named “revised manuscript with tracked change” and after careful reading, we found some response was inclompeted. To clarify the response, we have rewrite and add explanation on Reviewer #2:

Dear Editor,

Thank you very much for the opportunity to review this manuscript, that the topic is close to my research interest and geographic expertise. The authors have provide quite comprehensive study about the adherence of antenatal care in Semarang Indonesia.

From me, there are couple of minor revisions that the authors need to address before the publication of this paper:

The author mentions BemNOC many times, which is I understand, However, I would suggest to use a more widely used Basic Emergency Obstetric and Newborn Care (BEmONC)-See UNFPA publication, to represent Puskesmas with PONED-an Indonesian term for it.

Our response

Thank you for your suggestion regarding the acronym BEmONC. We have changed it throughout the manuscript as suggested.

2. Couple of abbreviations are not mentioned earlier but at the later part of the manuscript, for example LMIC-should be expand for the first time it is mentioned and BPJS-BPJSK.

Our response

Thank you for your correction. We have revised the word “BPJS to BPJS-K” throughout the manuscript as suggested

3. Details for data analysis, data collection, trustworthiness, demographic of qual participants for the qualitative interviews/FGs are missing.

Thank you for your careful review, we have added the data analysis on page 9 line 203-216

Data collection in. page 9 line 191-196

And demographic of qual participant as follows:

The demographic data of participant in in-depth interview of this study were :

| **Participant** | Participant 1 | Participant 2 | Participant 3 | Participant 4 | Participant 5 |
| --- | --- | --- | --- | --- | --- |
| **Age (year)** | 43 | 48 | 37 | 38 | 35 |
| **Working duration** | 22 | 24 | 15 | 17 | 15 |
| **Workplace** | PHC A | PHC B | PHC C | PHC D | PHD A |
| **Title** | midwife coordinator | midwife coordinator | midwife coordinator | midwife coordinator | Laboratory analyze |
| **Employment status** | permanent | permanent | permanent | permanent | permanent |

4. I would comment more also for the discussion and limitations, including implications for further research. This research would imply many, such as the need of training for non BEmONC, authority within primary care staff, and also perhaps further training for pregnancy complication screening-This was discussed superficially and authors discussed more on the numbers only.

Thank you for very careful review and also the suggestion sentences in the last part of discussion. We do agree to add more implication of our findings as follows:

The study revealed that healthcare providers in Semarang are not strictly adhering to the national integrated antenatal service guidelines. This is quite unexpected, especially since antenatal care coverage is as high as 98%. It is therefore crucial to reevaluate the criteria used to measure the success of ANC implementation, and not just focus on the quantity of care provided but also on its quality. These findings further support the research conducted in Tanzania, which revealed that the provision of ANC services was inconsistent and did not align with the recommended Focus Antenatal Care (FANC) guidelines^38^. (page 18-19, line 368-375)

We also added your suggestion in conclusion.

This study recommends the need for regionalization of PHC work area, the need of training for non BEmONC including training of pregnancy complication screening, authority within primary care staff, and also intra- and inter-health centers collaborative care to provide the high quality of integrated antenatal care for every woman in Indonesia. (Page 22 line 466-470)

5. Implication for further research and limitation of the study, including limited number of women and health providers involved in the study have not been clearly acknowledged..
Thank you for pointing this out, I would improve the implication of the study and include your suggestion, in term of sample size, I agree with you that the we included limited number of participant, however the sample size has met the minimum number according to Hosmer-Lameshow formula.

While Semarang is one of the big cities in Indonesia that usually the availability at least one doctor in the daily PHC practice is usually warranted. It raised concerns on the quality of care rather than numbers.

Thank you for pointing this out, we have included your suggestion in the discussion section.

Thank you very much.

[NOTE: If reviewer comments were submitted as an attachment file, they will be attached to this email and accessible via the submission site. Please log into your account, locate the manuscript record, and check for the action link "View Attachments". If this link does not appear, there are no attachment files.]

While revising your submission, please upload your figure files to the Preflight Analysis and Conversion Engine (PACE) digital diagnostic tool, <https://pacev2.apexcovantage.com/>. PACE helps ensure that figures meet PLOS requirements. To use PACE, you must first register as a user. Registration is free. Then, login and navigate to the UPLOAD tab, where you will find detailed instructions on how to use the tool. If you encounter any issues or have any questions when using PACE, please email PLOS at [figures@plos.org](mailto:figures@plos.org). Please note that Supporting Information files do not need this step.
